# Analysis of clinical patient-specific pre-treatment quality assurance with the new helical tomotherapy platform, following the AAPM TG-218 report

**DOI:** 10.1186/s13014-021-01952-w

**Published:** 2021-11-22

**Authors:** Marco Fusella, Samuele Cavinato, Alessandra Germani, Marta Paiusco, Nicola Pivato, Marco Andrea Rossato, Anthony Scott, Alessandro Scaggion

**Affiliations:** 1 Medical Physics Department, Veneto Institute of Oncology IOV-IRCCS, Via Gattamelata 64, 35128 Padova, Italy; 2 Dipartimento di Fisica e Astronomia ‘G. Galilei’, Università degli Studi di Padova, Padova, Italy; 3 The Abdus Salam International Centre for Theoretical Physics, Strada Costiera 11, 34151 Trieste, Italy

**Keywords:** TomoTherapy, Quality assurance, Pre-treatment quality assurance, IMRT QA

## Abstract

**Purpose:**

This study presents patient-specific quality assurance (QA) results from the first 395 clinical cases for the new helical TomoTherapy® platform (Radixact) coupled with dedicated Precision TPS.

**Methods:**

The passing rate of the Gamma Index (GP%) of 395 helical QA of patient-specific tomotherapy, acquired with ArcCHECK, is presented, analysed and correlated to various parameters of the plan. Following TG-218 recommendations, the clinic specific action limit (AL_cs_) and tolerance limit (TL_cs_) were calculated for our clinic and monitored during the analysed period.

**Results:**

The mean values ​​(± 1 standard deviation) of GP% (3%/2 mm) (both global and local normalization) are: 97.6% and 90.9%, respectively. The proposed AL_cs_ and TL_cs_, after a period of two years’ process monitoring are 89.4% and 91.1% respectively.

**Conclusions:**

The phantom measurements closely match the planned dose distributions, demonstrating that the calculation accuracy of the new Precision TPS and the delivery accuracy of the Radixact unit are adequate, with respect to international guidelines and reports. Furthermore, a first correlation with the planning parameters was made. Action and tolerance limits have been set for the new Radixact Linac.

## Introduction

In early 2018 a new version of the TomoTherapy® platform was commercially released (Radixact, Accuray, Sunnyvale, CA). This new machine is characterized by a higher dose rate of 1000 cGy/min, improved jaw dynamics, faster ring rotation (up to 10 rotations per minute), couch movement with separated axis and a couch catcher to reduce couch sag effect (to less than 2 mm) toward the gantry. The new platform comes with a redesigned version of the TPS: Precision TPS v1.0.02 which incorporates the graphics processing unit (GPU) based optimizer, VoLO™ and a Collapsed Cone Convolution Superposition dose calculation algorithm [1, 2].

This new system grants the capability of faster treatments and more freedom in the combination of the modulation factor, jaw width, pitch and gantry period. Thanks to these advanced features, the use of this system opens to the possibility of achieving more modulated and complex plans.

The long term dosimetric stability of this new delivery system has already been studied and reported by Smilowitz [3] and the new TPS version has been validated by Chen [2]. In order to thoroughly assess the new capability of the system, a detailed and complete analysis on a large database of clinical plans is also needed.

At our department, a Radixact unit machine has become clinical in June 2018 and since the first clinical plan a detailed QA program has been implemented following the recommendations of AAPM TG-218 [4]. Approximately 395 clinical helical plans were delivered and measured with ArcCHECK™ (Sun Nuclear, Melbourne, FL). Measured and calculated dose distributions were compared by means of 2D gamma analysis.

Within this work, we propose the clinic and machine specific tolerance limit (TL_cs_) and action limit (AL_cs_), obtained through statistical process control techniques, to be adopted for the new Radixact platform coupled to the Precision TPS v1.0.02. We also analyse and report which planning parameters are more related to QA failures.

## Materials and methods

### Database

Between June 2018 and October 2020 a total of 395 treatments have been planned with the Precision TPS v1.0.02 using the GPU-based optimizer VoLO™, which incorporates the Collapsed Cone Convolution Superposition dose calculation algorithm [1, 2]. The treatments were related to a large number of different sites and diseases; therefore, within the scope of this work they have been grouped in the following categories: abdomen (28 pts), brain (32 pts), head and neck (77 pts), lungs (71 pts), pelvis (84 pts), prostate (52 pts) and others (41 pts). The dose per fraction (D/fr) ranged from 1.6 to 5 Gy.

### Plans characteristics

To characterize the plans, the following parameters were collected: actual modulation factor (MF), pitch, field width (FW), gantry period, total delivery time, couch speed, couch travel distance and TTDF (ratio between the treatment time and the prescribed dose per fraction). Moreover, some descriptors of the leaf open time (LOT) distribution were collected: minimum (min-LOT), mean (mean-LOT), maximum (max-LOT) and LOT standard deviation (SD-LOT). All treatments utilized dynamic jaws [5].

### QA delivery and analysis

All the patient-specific QA measurements were collected with ArcCHECK™ (Sun Nuclear, Melbourne, FL) and performed without the 15 cm diameter homogeneous PMMA plug. The plans were recomputed on the synthetic ArcCHECK™ CT provided by the vendor with an imposed density of 1.1836 g/cm^3^ following manufacturer’s recommendations. Acquisition and analysis were performed with Sun Nuclear SNC Patient™ version 6.7. The absolute dose calibration of ArcCHECK™ was periodically controlled against a calibrated farmer-type ionisation chamber. Before each measurement session, the output of the Radixact unit was checked using the cheese phantom and ionization chambers, and a correction applied to the ArcCHECK™ analysis. QA plans were computed in high resolution mode which grants a 1.87 mm spaced dose grid.

The QA plan was computed placing the centre of the planning PTV at the machine’s isocenter. This procedure reduces the possibility for high dose areas to fall at the border of the ArcCHECK™ limiting the dependence of the gamma passing rate (GP%) on the maximum calculated dose [6].

According to the AAPM TG-218 report, the dose comparison was performed through 2D gamma analysis using a 10% dose threshold as well as 3% dose difference (DD) and 2 mm distance-to-agreement (DTA) criteria. In order to compare our results with previous works [5–8], 3%/3 mm gamma analysis was also collected. Both local and global normalizations were used.

### QA program

Following the AAPM TG-218 report, the clinical deliverability of a plan was evaluated on the basis of the universal tolerance limit (TL) and action limit (AL), which are 95% and 90% respectively, on the GP% computed with (3%, 2 mm), global normalization criteria.

Statistical process control techniques recommended in TG-218 have been employed to define a clinic specific TL_cs_ and AL_cs_ [4] as follows:

$${AL}_{cs}=100-3\sqrt{{\sigma }^{2}+{\left( \underset{\raise0.3em\hbox{$\smash{\scriptscriptstyle-}$}}{x} -100\right)}^{2}}$$

$${TL}_{cs}= \underset{\raise0.3em\hbox{$\smash{\scriptscriptstyle-}$}}{x} -2.660\underset{\_}{mR}$$

where $$\underset{\raise0.3em\hbox{$\smash{\scriptscriptstyle-}$}}{x}$$ is the GP% average over the investigation period, $$\sigma$$ is its standard deviation and $$\underset{\raise0.3em\hbox{$\smash{\scriptscriptstyle-}$}}{mR}$$ the moving average. These values were computed on the first 40 collected measurements. The IMRT treatment process has been monitored and investigated by periodically computing these values to verify whether the process was under control. For the subsequent evaluations, periods of approximately 6 months were considered. Only clinically deliverable plans, those fulfilling GP% (3%G, 2 mm) > 90%, were considered in this analysis and 95% confidence intervals on TL_cs_ and AL_cs_ have been computed through a bootstrap approach with 10,000 samples [9, 10].

### Statistical analysis

To spot possible differences of GP% among the different treatment sites a Kruskal-Wallis test was performed followed by a post-hoc analysis where Bonferroni correction was applied [11].

N-way analysis of variance (n-way ANOVA) was performed to spot the influence of the planning parameters on GP% [12]. Only parameters that strictly bore no correlations among themselves were included in the test (*p* > 0.05 and/or Pearson’s r < 0.8) [13, 14].

All tests were performed using MATLAB® R2020b (MathWorks, Inc., Natick, MA, USA). All the *p*-values reported are two-sided and *p* < 0.05 is considered as statistically significant.

## Results

**Fig. 1:**
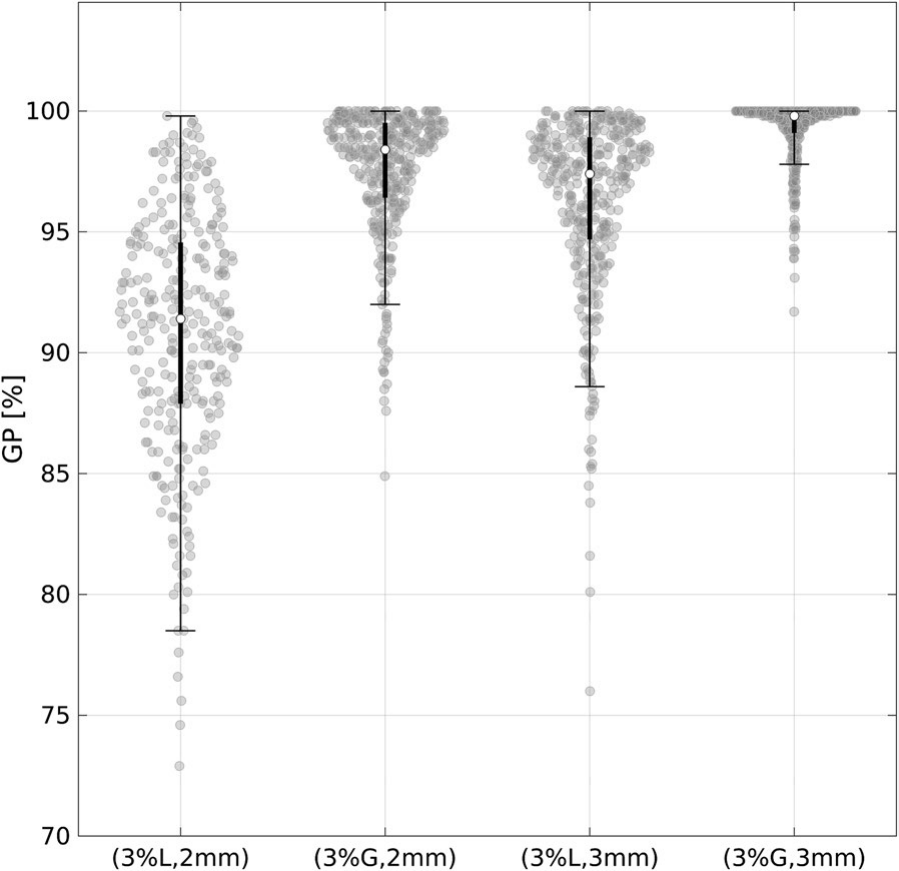
Violin plot of the complete database of collected measurements distinguished among the different gamma analysis criteria. The central white dot marks the median, the edges of the box correspond to the 25th and 75th percentiles, the whiskers extend to the adjacent values which are the most extreme data values that are not outliers, and data outside the whiskers are outliers. L stands for local gamma normalization, G for global one

Over the entire collection period, only 10 out of 385 plans failed to meet the universal AL defined by the TG-218 report. These failing plans were re-planned to meet the universal AL and added to the original set, thus resulting in a total of 395 plans that were actually measured. Figure 1 depicts the whole population of collected QA results.

**Fig. 2:**
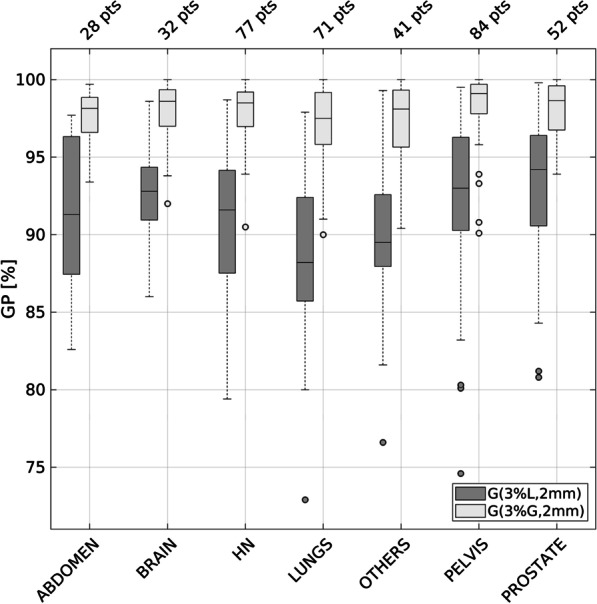
Whiskers box plot of GP% (3%, 2 mm) (global and local normalizations) of all measured plans distinguished among the different treatment sites. The central line marks the median, the edges of the box are the 25th and 75th percentiles, the whiskers extend to the adjacent values, which are the most extreme data values that are not outliers, and the circles represent the outliers

The measured 3%/2 mm GP% (both global and local normalizations) of the whole population (n = 395) was distinguished among treatment sites and the information is presented in Fig. 2. Data include both clinically and non-clinically (GP% (3%G, 2 mm) < 90%) delivered plans. The Kruskal-Wallis test confirmed that some differences among the groups does exist (*p*-value < 0.001). The post-hoc test showed that these differences are statistically significant only when the pelvis plans are compared to abdomen, lungs and others groups.

Table 1 reports the distribution of the collected planning parameters for our database of plans.

**Table 1:** Collected planning parameters for the entire population of 395 collected QA deliveries

	Average ± SD	Range [min; max]
Dose/Fraction [cGy]	219.8 ± 62.6	[160.0; 500.0]
MF	1.68 ± 0.28	[1.10; 2.79]
Pitch	0.39 ± 0.06	[0.12; 0.48]
Gantry Period [s]	20.1 ± 7.3	[11.8; 52.8]
Total Treatment Time [s]	299.7 ± 141.0	[66.3; 1241.3]
TTDF [s/cGy]	1.41 ± 0.69	[0.36; 5.43]
min-LOT [ms]	18.2±1.1	[18.0; 36.4]
mean-LOT [ms]	236.3 ± 82.7	[57.8; 617.1]
max-LOT [ms]	391.4 ± 144.0	[123.2; 1034.2]
SD-LOT [ms]	107.9 ± 44.2	[21.1; 316.1]
Couch speed [mm/s]	0.56 ± 0.20	[0.11; 1.84]
Couch travel distance [mm]	160.2 ± 88.9	[31.1; 895.2]
FW (10 mm/25 mm/50 mm)	2.1%/92.0%/5.9%	

**Fig. 3:**
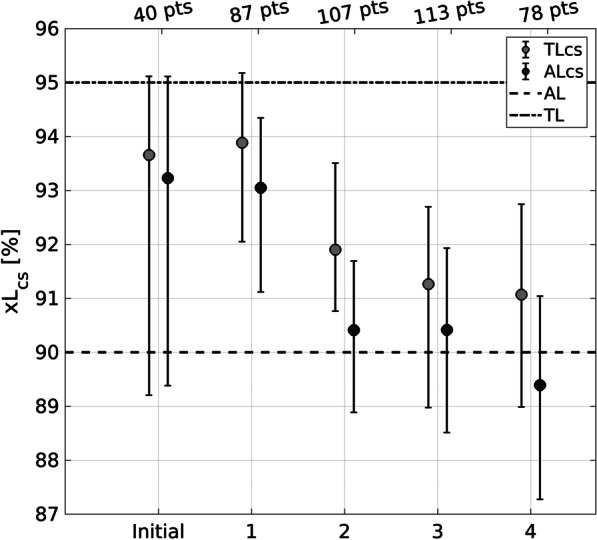
Evaluation of the AL_cs_ and TL_cs_. The circles mark the obtained values while the whiskers extend to the 95% confidence interval

Figure 3 reports the periodical evaluation of the AL_cs_ and TL_cs_; non-clinical plans are not included. The first 40 plans used for the first calculations (as proposed in TG-218), are also included in period “1”.

Among the first 40 planned treatments, the calculated AL_cs_ and TL_cs_ are, respectively, 93.2% and 93.7%, with all the plans satisfying GP% (3%G, 2 mm) > 93.3%. The final computation of AL_cs_ and TL_cs_ on the last set of 78 clinical plans yielded AL_cs_ = 89.4% and TL_cs_ = 91.1%.

A Kruskal-Wallis test has been performed to compare AL_cs_ and TL_cs_ samples of period 2, 3 and 4 and no statistically significant differences exist. In these three periods not even a single plan lies below the estimated AL_cs_, following the recommendations of TG-218, these evidences allow to state that the process is settled and under control. Using data from these 3 periods a n-way ANOVA was performed to detect which parameters might predict QA failures are summarized for both local and global normalizations of GP% with 3%/2mm criteria. Results are given in Table 2.

In Table 2, the results of the n-way ANOVA performed The analysis has been conducted for the data of the last three periods, where the process seems to become settled and under control (Fig. 3).

**Table 2:** Results of the n-way ANOVA test on GP% (3%, 2 mm) with respect to all the collected planning parameters in terms of *p*-values

	(3%L, 2 mm)	(3%G, 2 mm)
Site	0.002	0.063
MF	0.294	0.199
TTDF	0.007	0.373
mean-LOT	0.642	0.437
max-LOT	0.804	0.859
Pitch	0.884	0.659
Gantry period	0.820	0.841
Couch speed	0.189	0.055

Underlined values mark significant tests

## Discussion

In this work, we assessed the performances of the TomoTherapy® platform coupled with a new dedicated TPS in terms of the gamma index passing rate measured with a 3D dosimeter, extending the analysis of previous works on earlier versions of TomoTherapy® and associated TPS [7, 8]. The entire scope of work was performed following AAPM TG-218 recommendations. We considered the GP% metric with 3%/2 mm criteria, analysing also the impact of local and global normalizations. Our results showed that the mean GP% (3%G/2 mm) was 97.6 ± 2.6% with some differences arising among the different sites used to stratify the sample.

Any previous study on the TomoTherapy® unit has not reported results using the GP% metric defined by AAPM TG-218. For a comparison with existing literature we used the GP% with 3%/3mm criteria. The results here reportedly outperformed those obtained with different versions of tomotherapy machines and the same 3D dosimeter (ArcCHECK™) in terms of GP%. We measured systematically higher mean values with respect to Bresciani [7], Binny [15] and Yue [16]: 99.2% (SD 1.3%) vs. 96.1% (SD 4.4%), 95.9% (SD 2.9%) and 97.5% (SD 2.7%), respectively.

Applying the statistical process control techniques proposed by the Task Group, we periodically computed the centre-specific action level and tolerance limit (see Fig. 3). The first evaluation yielded values comparable to the universal ones indicated in the report (93.7% and 93.2%, versus 95% and 90% respectively). The periodical evaluation of the two control limits showed that a change occurred in the process between the first and the second re-evaluation. In fact, both TL_cs_ and AL_cs_ decreased and remained approximately constant afterwards (Fig. 3). This probably happened since the start of 2019 and after six months of initial training, new treatments have been introduced into the clinical practice (e.g. mesotheliomas, craniospinal irradiations, Hodgkin’s and non-Hodgkin’s lymphomas) that can be considered as inherently more complex; moreover, moving up the learning curve, the planners have begun to achieve increasingly complex and modulated dose distribution; thus probably inducing a decrease of treatment deliverability. All this sources of variation forced to follow TG-218 recommendations, going through a periodically monitoring of the process, investigating the GP% over a long period.

To spot possible planning parameters closely related to QA results, and their trend, a n-way ANOVA test was performed (see Table 2). Only the treatment site and the TTDF (treatment time divided by the prescribed dose per fraction) were highlighted as possible predictors of QA failures, only when GP% (3%L,  2mm) is considered. In fact, the TTDF can be considered as a simple indicator of complexity, since it is somehow related to the longitudinal extension of the target volume and to the pitch. A similar behaviour was recently reported by Santos et al. [17].

Previous studies reported the effect of mean-LOT, max-LOT, MF, pitch and gantry period on plan deliverability [6, 7, 15, 18]. In our database, none of the collected parameter can be considered relevant when the GP% (3%G, 2 mm) is used, and this might be due to different reasons. First of all, the presence of the TTDF might mask all the other parameters, since mathematical relationships hold between the TTDF and most of the aforementioned parameters. Secondly, our planning practice strictly follows the recommendation of the Accuray planning manual [1] which suggests to prepare plans with max-LOT higher than 241 ms, and mean-LOT higher than 100 ms. Moreover, approximately 95% of our plans show the MF within 1.4 and 2.5, which Binny et al. [18] have previously shown to be associated to acceptable deliverability.

Because no dependence from anatomical site has been found for GP% (3%G/2 mm), it is possible to use for all treatments the same AL_cs_ and TL_cs_ here reported. When the more stringent local criteria is considered a significant difference among the anatomical site arouse. In this case group-specific AL_cs_ and TL_cs_ would have been required and, following AAPM TG-218 recommendation, should be computed.

The assessment of the TPS’s performances in regards to complexity of the calculated sinograms is beyond the scope of this work, as well as the detailed analysis of the relationship between GP% and clinically relevant deviations [6, 19–21]. A more detailed analysis on the predictive value of the analysed parameters on QA trend and failures is still ongoing.

The results of this study highlight the capability of the Radixact system to accurately deliver complex dose distributions over a large variety of treatment sites. We set the TL and AL for this new machine to be used as reference or comparison for other centres.

## Conclusions

The results presented in this study suggest that the calculation accuracy of the new Precision TPS and the delivery accuracy of the Radixact unit is adequate, with respect to international guidelines and reports. Following the TG-218 methodology, we calculated both tolerance and action limits for the determination of the clinical deliverability of plans calculated with the new TPS, Accuray Precision, for the new TomoTherapy® version, (Radixact). These values have been monitored over a long period, and can be used as reference or comparison for other centres implementing this new technology.

## Data Availability

Not applicable.

## Abbreviations

AL: Action limit
AL_cs_: Center specific action limit
CT: Computed tomography
DD: Dose difference
DTA: Distance-to-agreement
FW: Field width
GP%: Gamma-analysis passing rate
LOT: Leaf open time
MF: Actual modulation factor
PTV: Planning target volume
QA: Quality assurance
TG: Task group
TL: Tolerance limit
TL_cs_: Center specific tolerance limit
TPS: Treatment planning system
TTDF: Treatment time over dose per fraction
